# Clinical Switching Strategies of Various Antidepressants to Vortioxetine in the PREDDICT Trial

**DOI:** 10.1093/ijnp/pyaa092

**Published:** 2020-12-03

**Authors:** Natalie T Mills, Emma Sampson, Célia Fourrier, Bernhard T Baune

**Affiliations:** 1 Discipline of Psychiatry, Adelaide Medical School, University of Adelaide, Adelaide, Australia; 2 Hopwood Centre for Neurobiology, Lifelong Health Theme, South Australian Health and Medical Research Institute, Adelaide, Australia; 3 Department of Psychiatry and Psychotherapy, University Hospital Münster, University of Münster, Münster, Germany; 4 Department of Psychiatry, Melbourne Medical School, The University of Melbourne, Melbourne, Australia; 5 The Florey Institute of Neuroscience and Mental Health, The University of Melbourne, Parkville, Australia

**Keywords:** Major depressive disorder, vortioxetine, cross-titration, side-effects, switching

## Abstract

**Background:**

Partial response to antidepressant medication as well as relapse and treatment resistance are common in major depressive disorder (MDD). Therefore, for most patients with MDD, there will be a need to consider changing antidepressant medication at some stage during the course of the illness. The PREDDICT study investigates the efficacy of augmenting vortioxetine with celecoxib.

**Methods:**

We describe the method used in the PREDDICT study to change participants, who were already taking antidepressant medication at the time of the screening visit, to vortioxetine. We used a cross-titration to change study participants to vortioxetine.

**Results:**

Of a total of 122 study participants who were randomized to receive vortioxetine plus celecoxib or vortioxetine plus placebo at the study baseline visit, 82 were taking antidepressant medication (other than vortioxetine) prior to randomization. These medications were selective serotonin reuptake inhibitors, serotonin noradrenaline reuptake inhibitors, tricyclic antidepressants, mirtazapine, or agomelatine. Eighty of these 82 participants completed the changeover to vortioxetine as well as the study baseline visit. We found side effects were generally mild during this changeover period. In addition, there was a reduction in mean total Montgomery-Åsberg Depression Rating Scale score of 2.5 (SD 6.0) from study baseline to week 2 and a further reduction in mean total Montgomery-Åsberg Depression Rating Scale of 2.5 (SD 5.9) from week 2 to week 4.

**Conclusion:**

Changing other antidepressants to vortioxetine can be done safely and was generally well-tolerated. However, there are some antidepressant classes, in particular monoamine oxidase inhibitors that require a washout period, which were not represented in this study.

**Trial registration:**

Australian New Zealand Clinical Trials Registry (ANZCTR); ID number 12617000527369p; http://www.anzctr.org.au/ACTRN12617000527369p.aspx

**Significance Statement**

Many patients suffering from major depressive disorder (MDD) will need to consider, in conjunction with their treating doctors, changing antidepressant medication at some stage during the illness. Choosing a clinically suitable change-over strategy is crucial for achieving efficacy, reducing potential withdrawal effects from the previous antidepressant, and minimizing side effects of the new antidepressant. To our knowledge, such a strategy has not been systematically investigated for the newest available antidepressant vortioxetine under real-world conditions. Here we describe the results of a clinical trial that employed various change-over strategies for commonly used antidepressants showing that the change-over strategies to vortioxetine were safe and generally well-tolerated while achieving efficacious treatment outcomes.

## Introduction

Major depressive disorder (MDD) is a serious problem worldwide, with chronic illness common (Whiteford et al., 2013). With the illness often characterized by recurrent episodes (Trivedi et al., 2006), there is also marked impairment of functioning (McKnight and Kashdan, 2009). Furthermore, only approximately one-third of patients achieve remission with the first antidepressant treatment (Trivedi et al., 2006), and treatment resistance is common (Rush et al., 2006).

Vortioxetine is a novel multi-modal antidepressant (Katona and Katona, 2014; Sanchez et al., 2015). In addition to inhibition of the serotonin transporter, it has effects on several serotonin receptors (Katona and Katona, 2014; Sanchez et al., 2015). Specifically, vortioxetine has been found to display 5-HT_3_ and 5-HT_7_ antagonism, partial agonist properties at 5-HT_1B_ receptors, agonist properties at 5-HT_1A_ receptors, and potent inhibition of the serotonin transporter (Bang-Andersen et al., 2011). Vortioxetine has a long half-life of approximately 66 hours (Chen et al., 2018), which is thought to at least partly explain its low rate of withdrawal or discontinuation symptoms (Renoir, 2013; Sanchez et al., 2015).

Vortioxetine has been found to have efficacy in the treatment of MDD as well as in the prevention of relapse (Boulenger et al., 2012; Katona and Katona, 2014). Efficacy of vortioxetine vs placebo in treating MDD has also been demonstrated by meta-analyses (Pae et al., 2015; Thase et al., 2016), including a treatment effect increasing with dose (from 5 mg to 20 mg daily) of vortioxetine (Thase et al., 2016). Vortioxetine has also been observed to improve the cognitive symptoms associated with MDD (Katona et al., 2012; Al-Sukhni et al., 2015; Mahableshwarkar et al., 2015a; Kennedy et al., 2016; McIntyre et al., 2016; Baune et al., 2018). Using the digit symbol substitution test, a recent network meta-analysis found vortioxetine to be the only antidepressant with greater efficacy than placebo in improving this measure of cognitive dysfunction in MDD (Baune et al., 2018). Furthermore, vortioxetine is generally well-tolerated (Cipriani et al., 2018), providing further rationale for choosing it in this study.

Some studies using lower doses of vortioxetine have found no difference from placebo. Specifically, a 6-week, randomized controlled, double blind trial found no significant difference between vortioxetine 5 mg daily and placebo in adults with MDD (Jain et al., 2013), and a randomized double blind study using duloxetine as a reference found no significant difference between vortioxetine 15 mg daily and placebo, but vortioxetine 20 mg daily was superior to placebo (Mahableshwarkar et al., 2015b).

Changing antidepressants can be associated with worsening of mood, in particular if there is a washout period (Keks et al., 2016), as well as withdrawal or discontinuation symptoms. If there is a crossover period, where the new antidepressant is commenced at a low dose while the dose of the prior antidepressant has been reduced (but not yet ceased), then there is a risk of additional side effects, including serotonin syndrome (Keks et al., 2016). Hence there is a rationale for tapering the first antidepressant, with a washout period before commencing the alternative antidepressant, and a different rationale for using a crossover when changing antidepressants.

The 2016 Canadian Network for Mood and Anxiety Treatments clinical guidelines for MDD recommend, if possible, a slow tapering of antidepressants over several weeks (Kennedy et al., 2016). Directly switching vs a crossover/cross-titration of a selective serotonin reuptake inhibitor (SSRI) to duloxetine has been investigated in terms of both efficacy and tolerability (Perahia et al., 2008). Specifically, the authors found that both methods, that is, ceasing the SSRI and commencing duloxetine immediately afterwards, or using a cross-titration of antidepressants, were both well-tolerated, with a significant improvement in depressive symptoms (Perahia et al., 2008). In summary, it is not clear whether directly switching or cross-titration works best when changing antidepressants, in particular when switching to the relatively new antidepressant vortioxetine.

The PREDDICT study investigates the efficacy of augmenting vortioxetine with the anti-inflammatory celecoxib in MDD (Fourrier et al., 2018). Study participants met the criteria for current MDD at the time of screening. As study participants were already experiencing significant depressive symptoms at the time of commencing the study, a cross-titration method was used to change to vortioxetine. In addition, as only one-half the study participants who were taking an antidepressant at screening were changing from an SSRI to vortioxetine, we chose cross-titration rather than direct switch. Here, we describe in more detail this cross-titration method that we used for the PREDDICT study. We also describe side effects experienced by study participants during this cross-titration period and during the first 4 weeks of the randomized controlled phase of the study. In addition, we use Montgomery-Åsberg Depression Rating Scale (MADRS) scores from the first 4 weeks of the randomized controlled phase to compare time to efficacy of vortioxetine (in study participants undergoing the cross-titration with those who were not taking antidepressant medication at the study screening visit). Results from the end of the randomized controlled phase will be reported at a later date (as a main outcome of the PREDDICT study).

Therefore our objectives are to (1) present the cross-titration method used in PREDDICT; (2) describe side effects from vortioxetine, with those participants on no antidepressant at time of screening as a comparison group; and (3) compare time to onset of efficacy of vortioxetine between those participants cross-titrating from a previous antidepressant vs those with no antidepressant at screening.

## Methods

The PREDDICT study is an 8-week randomized controlled trial (RCT) plus 6-month post-RCT follow-up period investigating the short-term efficacy of augmenting vortioxetine with celecoxib in MDD (Fourrier et al., 2018) as well as the longer term effects of vortioxetine (without celecoxib) on cognitive function, psychosocial function, quality of life, and workplace functioning. The study protocol has been described in detail elsewhere, with the screening visit including an assessment for MDD as well as depressive symptom severity (Fourrier et al., 2018). Depressive symptom severity was assessed with the MADRS. At the screening visit, study participants were also asked about antidepressant medication (both during the current major depressive episode and previous episodes), including the length of time these antidepressants were taken for and whether the participant found each medication to be effective. The Royal Adelaide Hospital Pharmacy generated a computer-sequenced randomization table (to celecoxib or placebo) with an arm for study participants with C-reactive protein (CRP) ≤ 3 mg/L at screening and an arm for participants with CRP > 3 mg/L at screening. The trial psychiatrist followed the sequence of the randomization table in date order of study baseline visit (with CRP at screening determining which of the arms [CRP ≤ 3 mg/L or CRP > 3 mg/L] in the randomization table to go to). PREDDICT was approved by CALHN Human Research Ethics Committee (HREC/17/RAH/111, CALHN reference no. R20170320) and the University of Adelaide Human Research Ethics Committee. Study participants provided informed written consent. The study was also registered with the Australian and New Zealand Clinical Trials Registry prior to enrolment of the first study participant (Australian and New Zealand Clinical Trials Registry Registration no. 12617000527369p).

**Figure 1.**
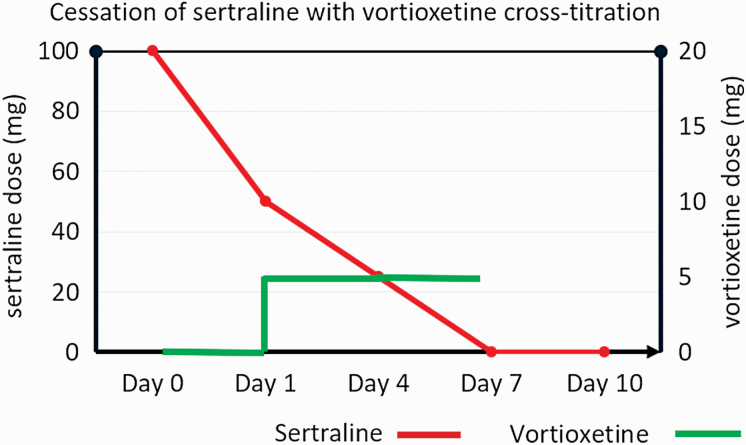
Example of cross-titration from the selective serotonin reuptake inhibitor (SSRI) sertraline to vortioxetine. At baseline visit, study participants randomized to celecoxib continued vortioxetine at 5 mg daily and study participants randomized to placebo increased to vortioxetine 10 mg daily.

Study participants who were taking a different antidepressant than vortioxetine participated in a “pre-baseline” visit, where advice was given by the trial psychiatrist on the cross-titration process. For study participants taking SSRIs, participants first halved the dose of the SSRI and then commenced vortioxetine at 5 mg daily on the same day. After a further 3 days, the SSRI dose was halved again. This was continued (with the SSRI dose halved approximately every 3 days) until the SSRI was at a dose where it could be ceased (see Figure 1 for an example; in this example, the study participant would return for the study baseline visit on day 8). Vortioxetine was continued at 5 mg daily during this time.

Similarly, for serotonin noradrenaline reuptake inhibitors (SNRIs), once a study participant had halved the dose, vortioxetine was commenced at 5 mg daily. After 3–4 days, the dose of the SNRI was halved again. This continued until the study participant was taking the lowest possible daily dose of the SNRI (venlafaxine XR 37.5 mg daily, desvenlafaxine 25 mg daily, or duloxetine 30 mg daily) before it was ceased in the next step. To try to minimize discontinuation symptoms, when study participants were on the lowest dose of an SNRI (e.g., 37.5 mg daily for venlafaxine XR), the SNRI was taken on alternate days for 3–4 days before ceasing. Vortioxetine continued at 5 mg daily during this period, and once the SNRI was ceased, study participants attended for the baseline visit. There were some exceptions to this, in particular for study participants taking venlafaxine XR 300 mg daily, where a longer time frame was used to reduce to one-half of the initial antidepressant dose (study participants remained on venlafaxine XR 225 mg daily for at least 3–4 days before decreasing any further). With initial higher doses of SNRIs, study participants did not commence vortioxetine 5 mg daily until the SNRI was reduced by at least 50% of the initial dose.

Tricyclic antidepressants and mirtazapine were changed over in a similar way to the SSRIs. If a study participant was taking a low dose of mirtazapine and this was needed to assist with sleep, then this could be continued during the study. Monoamine oxidase inhibitors (MAOIs) such as phenelzine and tranylcypromine require a washout period of 14 days (Keks et al., 2016); however, no study participants were taking a MAOI prior to the study baseline visit.

Agomelatine could be ceased without tapering. Therefore, study participants taking agomelatine (without another antidepressant) had the last dose of agomelatine the night before the baseline visit. Vortioxetine was then commenced the next day at the study baseline visit.

For those study participants taking 2 antidepressant medications prior to the pre-baseline visit, if 1 of these antidepressants was agomelatine, first this was ceased without tapering as described above. Then the other antidepressant medication was cross-titrated with vortioxetine in the same way as described above for the particular antidepressant class. For the other participants taking 2 antidepressants prior to the pre-baseline visit, 1 of these antidepressants was mirtazapine. Therefore, the other antidepressant was tapered and ceased first, then mirtazapine was reduced to a lower dose, and then vortioxetine 5 mg daily was commenced.

Participants were asked at the baseline visit about any side effects and antidepressant discontinuation symptoms they had experienced during the cross-titration period. We defined discontinuation symptoms as those described in the literature such as dizziness, shock-like sensations, or nausea (Black et al., 2000) that were also consistent with the time frame for withdrawal symptoms, that is, symptoms beginning within 1–7 days of dose reduction (Black et al., 2000). As nausea is one of the more common side effects of vortioxetine, if nausea was still present at the RCT week 2 visit, we classed this as a side effect of vortioxetine.

At the baseline visit, study participants randomized to celecoxib received vortioxetine 5 mg daily. If the study medication was well tolerated, vortioxetine could then be increased to 10 mg daily at the RCT week 2 visit, with potential to increase to vortioxetine 20 mg daily at the RCT week 6 visit. Vortioxetine was not increased further than 10 mg daily while celecoxib was co-administered, as it is advised to halve the dose of vortioxetine during co-administration with CYP2D6 inhibitors (Chen et al., 2018). Those study participants randomized to placebo at the baseline visit received vortioxetine 10 mg daily, which could be increased to 20 mg daily at the RCT week 2 visit (or at subsequent RCT visits if side effects had not settled by RCT week 2).

At all study visits after the baseline, participants were asked about side effects, including the emergence of new side effects, and were asked to rate each side effect as mild, moderate, or severe. Additional information such as the frequency of the side effect could also be written in the severity or duration column of the side effect scale (see supplementary Table 1 for the side effects scale used). To compare time to efficacy of vortioxetine, we compared the change in MADRS up to and including the RCT week 4 visit, as any delay in onset of efficacy in vortioxetine, as well as side effects, should have emerged by this time.

## Results

A total of 122 people were randomized to receive either vortioxetine plus celecoxib or vortioxetine plus placebo. Of these, 82 were taking antidepressant medication (other than vortioxetine) at the time of the PREDDICT screening visit. One study participant had commenced vortioxetine several months before the study baseline visit and was therefore not included in the cross-titration or comparison (no antidepressant at screening) group. One study participant had been randomized (had completed the changeover of medication to vortioxetine) but did not proceed with the study baseline visit due to a further lowering of mood. Another study participant had been randomized but advised the study team on the day of the baseline visit they had not commenced the changeover of medication to vortioxetine and did not wish to proceed. A further study participant commenced the changeover of antidepressant to vortioxetine but at day 4 of the 8 day cross-titration ceased vortioxetine and returned to the previous dose of antidepressant due to lowering of mood (therefore, this study participant was not randomized to receive celecoxib or placebo). Therefore, a total of 80 study participants completed the cross-titration of antidepressant medication plus the study baseline visit.

Table 1 shows antidepressant medication at the time of the study pre-baseline visit (where antidepressant is changed to vortioxetine) for those study participants who went on to be randomized to celecoxib or placebo and also completed the study baseline visit. The most common medications at the time of the pre-baseline visit were desvenlafaxine, sertraline, venlafaxine, and escitalopram. Other medications (in order of frequency) were duloxetine, fluoxetine, agomelatine, citalopram, mirtazapine, fluvoxamine, paroxetine, and dothiepin.

**Table 1.** Antidepressant Medications at PREDDICT Pre-Baseline Visit

Medication	No., participants	No., males	No., females	Age range (y)	Dose range (mg)
Sertraline	13	2	11	25–64	50–200
Fluoxetine	7	1	6	24–68	20–40
Fluvoxamine	3	1	2	19–64	50–150
Citalopram	4^*a*^	2^*a*^	2	41–64	20–80
Escitalopram	11	3	8	18–59	10–40
Paroxetine	2	2	0	47–51	20–60
Duloxetine	10	4	6	39–66	30–120
Desvenlafaxine	14	7	7	35–63	50–200
Venlafaxine	11^*b*^	5	6^*b*^	20–64	75–300
Agomelatine	6^*c*^	4^*c*^	2^*c*^	39–67	25–50
Mirtazapine	4	2	2	53–75	45–90
Dothiepin	1	1	0	55	150
TOTAL	80	31	49	18–75	—

^*a*^One study participant was taking citalopram and mirtazapine at the pre-baseline visit (this participant is counted only once in the “Total” row at the bottom of the table).

^*b*^One study participant was taking venlafaxine XR and mirtazapine at the pre-baseline visit (hence this study participant is counted only once in the “Total” row).

^*c*^Four study participants taking agomelatine (2 male and 2 female) were also taking another antidepressant; hence, these participants are counted only once in the “Total” row at the bottom of the table.

Six participants were taking 2 antidepressants at the time of the pre-baseline visit. Of these 6 study participants, 4 were taking agomelatine plus another antidepressant (desvenlafaxine or duloxetine). One study participant was taking a combination of venlafaxine XR and mirtazapine at the study pre-baseline visit, while another was taking a combination of citalopram and mirtazapine.

The majority (61.3%) of study participants who were changing antidepressant medication to vortioxetine (and then went on to complete the study baseline visit) were female (Table 1). Study participants covered the full age range specified in the PREDDICT protocol (18-75 years). There was a large variation in the amount of time study participants had been taking the current antidepressant medication. The shortest duration was 2 months, while the longest was 20 years.

The cross-titration period ranged from 1 day to 21 days, with study participants who were taking agomelatine alone not requiring a cross-titration (hence, agomelatine was ceased and vortioxetine commenced the next day at the study baseline visit). The longest changeover period was for venlafaxine XR, with doses of 300 mg daily requiring a 21-day cross-titration.

Table 2 shows side effects from vortioxetine experienced during the changeover of antidepressant (at time of baseline visit) to vortioxetine and during the first 4 weeks of the RCT phase. We found low rates of side effects during the cross-titration period, and these side effects were generally mild (Table 2). The most common side effect experienced during the cross-titration was nausea, which in most instances was mild and was helped considerably by taking vortioxetine with food (e.g., at the end of breakfast). Other gastrointestinal symptoms (diarrhea) were also mild (Table 2). Other side effects reported as experienced during the cross-titration (Table 2) were headaches (6 participants), sweating (3 participants), pruritis (2 participants), agitation (2 participants), and tiredness (1 participant). Table 2 also shows side effects that either emerged after the baseline visit (but before the RCT week 2 visit) or were still present at the week 2 or week 4 RCT visit. The corresponding numbers of study participants randomized to receive celecoxib and placebo are also shown in Table 2.

**Table 2.** Side Effects From Vortioxetine During the Cross-Titration Period and First 4 Weeks of the RCT Phase for Participants on an Antidepressant Prior to Baseline^*a*^

Side effect	% (n) Participants (out of 80)	% (n) Side effect emerged or still present at RCT2 (out of 79)	% (n) Celecoxib/ % (n) placebo at RCT2	% (n) Side effect still present at RCT4 (out of 73)	% (n) Celecoxib/% (n) placebo at RCT4
Nausea	23.8 (19)	21.5 (17)	11.3 (9)/ 10.1 (8)	9.6 (7)	5.5 (4)/4.1 (3)
Vomiting	0 (0)	1.3 (1)	0 (0)/ 1.3 (1)	0 (0)	n/a
Diarrhea	10.0 (8)	12.7 (10)	8.9 (7)/ 3.8 (3)	8.2 (6)	6.8 (5)/ 1.4 (1)
Headaches	7.5 (6)	8.9 (7)	6.3 (5)/ 2.5 (2)	2.7 (2)	2.7 (2)/ 0 (0)
Pruritis	2.5 (2)	7.6 (6)	3.8 (3)/ 3.8 (3)	5.5 (4)	1.4 (1)/ 4.1 (3)
Agitation	2.5 (2)	13.9 (11)	8.9 (7)/ 5.1 (4)	13.7 (10)	11.0 (8)/ 2.7 (2)
Tiredness	1.3 (1)	10.1 (8)	6.3 (5)/3.8 (3)	2.7 (2)	1.4 (1)/ 1.4 (1)
Sweats or flushes	3.8 (3)	5.1 (4)	5.1 (4)/ 0 (0)	4.1 (3)	4.1 (3)/ 0(0)

Abbreviations: n/a, not applicable; RCT, randomized controlled trial; RCT2, RCT week 2 visit; RCT4, RCT week 4 visit.

^*a*^A total of 43 participants were randomized to celecoxib, 37 were randomized to placebo.

Except for nausea, there were more study participants reporting each of the side effects listed in Table 2 at the RCT week 2 visit compared with the baseline visit (covering the cross-titration). Reasons for this could have included side effects from celecoxib, particularly with regard to diarrhea, or discontinuation symptoms, particularly with regard to agitation. For study participants randomized to receive celecoxib, the dose of vortioxetine remained at 5 mg daily for at least the first 2 weeks of the RCT.

As a comparison group, rates of side effects in those participants not taking antidepressant medication at the time of screening are in Table 3. The total number of participants experiencing side effects from the time of the baseline visit to the RCT week 4 visit are shown (column 2) as well as numbers of participants still experiencing these side effects at the week 2 or week 4 RCT visit (columns 4 and 6). The corresponding numbers of study participants randomized to receive celecoxib and placebo are also shown. Rates of reported tiredness were higher in those participants not taking antidepressant medication at the time of screening (*P* = .047 at RCT 2) as were rates of abdominal pain, headaches (*P* = .016 at RCT 2), and chest pain or discomfort. A greater percentage of participants on no antidepressant medication at screening reported nausea up to and including the RCT week 2 visit (*P* = .031); however, this group reported lower rates of nausea at the RCT week 4 visit (nausea reported by only 1 participant at RCT 4). Rates of diarrhea, agitation, and sweats or flushes were higher in the group of participants who cross-titrated antidepressant medication to change to vortioxetine. In both groups, a similar proportion of study participants reported pruritis at the RCT week 2 visit (7.6% in those who underwent cross-titration of antidepressant, 7.9% of participants with no antidepressant at screening).

**Table 3.** Side Effects From Vortioxetine During First 4 Weeks of RCT Phase in Participants Not Taking Antidepressant Immediately Prior to Baseline^*a*^

Side effect	% (n) Total Participants (out of 38)	% (n) Celecoxib/% (n) placebo	% (n) Side effect still present at RCT2 (out of 37)	% (n) Celecoxib/% (n) placebo at RCT2	% (n) Side effect still present at RCT4 (out of 36)	% (n) Celecoxib/% (n) placebo at RCT4
Nausea	44.7 (17)	10.5 (4)/ 34.2 (13)	27.0 (10)	8.1 (3)/ 18.9 (7)	2.8 (1)	0.0 (0)/ 2.8 (1)
Diarrhoea	7.9 (3)	0.0 (0)/ 7.9 (3)	2.7 (1)	0.0 (0)/ 2.7 (1)	2.8 (1)	0.0 (0)/ 2.8 (1)
Constipation	13.2 (5)	2.6 (1)/ 10.5 (4)	8.1 (3)	2.7 (1)/ 5.4 (2)	0.0 (0)	n/a
Abdominal pain	10.5 (4)	0.0 (0)/10.5 (4)	5.4 (2)	0.0 (0)/ 5.4 (2)	2.8 (1)	0.0 (0)/ 2.8 (1)
Headaches	28.9 (11)	5.3 (2)/ 23.7 (9)	13.5 (5)	2.7 (1)/ 10.8 (4)	5.6 (2)	2.8 (1)/ 2.8 (1)
Dizziness	15.8 (6)	5.3 (2)/ 10.5 (4)	8.1 (3)	2.7 (1)/ 5.4 (2)	2.8 (1)	2.8 (1)/ 0.0 (0)
Pruritis	15.8 (6)	7.9 (3)/ 7.9 (3)	16.2 (6)	8.1 (3)/ 8.1 (3)	5.6 (2)	0.0 (0)/ 5.6 (2)
Agitation	2.6 (1)	0.0. (0)/ 2.6 (1)	0.0 (0)	n/a	n/a	n/a
Tiredness	26.3 (10)	5.3 (2)/ 21.1 (8)	13.5 (5)	5.4 (2)/ 13.5 (5)	13.9 (5)	2.8 (1)/ 11.1 (4)
Chest pain/ discomfort	2.6 (1) (1/0)	2.6 (1)/ 0.0 (0)	0.0 (0)	n/a	n/a	n/a
Weight gain	2.6 (1)	0.0 (0)/ 2.6 (1)	2.7 (1)	0.0 (0)/ 2.7 (1)	0.0 (0)	n/a
Sexual dysfunction	2.6 (1)	2.6 (1)/ 0.0 (0)	2.7 (1)	2.7 (1)/ 0.0 (0)	0.0 (0)	n/a

Abbreviations: n/a, not applicable; RCT, randomized controlled trial; RCT2, RCT week 2 visit; RCT4, RCT week 4 visit.

^*a*^Sixteen participants were randomized to celecoxib, and 22 were randomized to placebo.

From the criteria we described in the Methods section to define discontinuation symptoms (those described in the literature such as dizziness, shock-like sensations, or nausea, beginning within 1–7 days of antidepressant dose reduction) (Black et al., 2000), 19 study participants experienced withdrawal or discontinuation symptoms in the cross-titration period (Table 4). These symptoms were generally mild (e.g., occasional “brain zaps”), which study participants reported as not troubling. These withdrawal or discontinuation symptoms were experienced by study participants who were changing from venlafaxine (4 participants), desvenlafaxine (8 participants), duloxetine (3 participants), paroxetine (1 participant), escitalopram (1 participant), citalopram (1 participant), and fluoxetine (1 participant). The most common discontinuation symptoms were mild “brain zaps” or a “buzzing” sensation in the head, or paresthesia (9 participants, with 1 of these participants reporting paresthesia of the hand; Table 4). Some study participants experienced more than 1 discontinuation symptom (as shown in Table 4). Two study participants changing from fluoxetine reported a lowering of mood during this time. No study participants experienced serotonin syndrome during the changeover to vortioxetine.

**Table 4.** Discontinuation Symptoms With Changeover to Vortioxetine

Discontinuation symptom	% (n) Participants (out of 80)	% (n) Participants with symptoms at RCT2 (out of 79)
Any symptom	23.8 (19)	3.8 (3)
“Brain zaps,” “buzzing,” or paresthesia	11.3 (9)	2.5 (2)
Nausea	1.3 (1)	0.0 (0)
Light-headedness	8.8 (7)	1.3 (1)
Tremor	1.3 (1)	0.0 (0)
Irritability	5.0 (4)	0.0 (0)
Headaches	3.8 (3)	0.0 (0)

Abbreviation: RCT2, randomized controlled trial week 2 visit.

For 3 participants who experienced discontinuation symptoms, these symptoms were still present at the RCT week 2 visit (Table 4). However, as these symptoms were typical of discontinuation symptoms and were improving, we classed these as discontinuation effects.

From the baseline visit to the RCT week 2 visit, we observed a mean reduction in total MADRS score of 2.5 (SD 6.0) in those study participants who had undergone a cross-titration from a previous antidepressant compared with a mean reduction in total MADRS of 4.5 (SD 6.4) in those participants who were not taking antidepressant medication at screening. From the RCT week 2 visit to RCT week 4, there was a further decrease in mean total MADRS for the group who had cross-titrated to vortioxetine of 2.5 (SD 5.9) and a mean decrease in total MADRS of 2.3 (SD 5.9) for those participants without antidepressant medication at screening. There was no significant difference in the change in MADRS between the 2 groups at the RCT week 2 visit (*P* = .11) or RCT week 4 visit (*P* = .97).

## Discussion

Of 122 study participants randomized to receive either vortioxetine plus celecoxib or vortioxetine plus placebo, 82 (67.2%) were taking an antidepressant medication prior to commencing the PREDDICT study. We used a cross-titration method to change study participants from their current antidepressant medication to vortioxetine. This method was generally well-tolerated; however, some study participants experienced a significant lowering of mood during this changeover period, with 2 study participants commencing the changeover to vortioxetine but not going ahead with the study baseline visit.

Side effects from vortioxetine were generally mild (as reported by study participants), with the most common side effects being nausea, diarrhea, and mild headaches in the group who cross-titrated antidepressant medication. By comparison, in the group of participants who were not taking antidepressant medication at time of screening, the most common side effects were nausea, headaches, and tiredness. In total, 36 of 118 study participants (30.5%) experienced nausea. This rate of nausea was within the range reported in previous clinical trials of vortioxetine, with rates of nausea of 8.8% to 38% reported in short-term trials and 8.8% to 31% reported in long-term trials (Al-Sukhni et al., 2015). In our study, for the group not taking antidepressant medication at screening, a higher number of participants were randomized to placebo (22 of 38 participants) rather than celecoxib. This could partly explain the higher rates of side effects such as nausea in this group, as study participants randomized to receive placebo were commenced on a dose of vortioxetine 10 mg daily (rather than 5 mg daily). It is also quite possible that a number of the side effects study participants described at the RCT week 2 and RCT week 4 visits were due to celecoxib, in particular the gastrointestinal side effects. Furthermore, some side effects at these visits could possibly be from placebo.

Withdrawal or discontinuation symptoms from the antidepressant that the study participants were changing from were also generally mild and short-lived. We found this appeared to be helped by commencing a low dose of vortioxetine (5 mg daily) so that study participants did not have a washout period. However, if there had been study participants commencing the changeover to vortioxetine from a MAOI, we would have had to use a washout period of 14 days (and allowed longer for the changeover to vortioxetine).

We could also have used a washout period; however, as study participants were already experiencing significant depressive symptoms at the time of screening, we chose the cross-titration method. A randomized double-blind study switched adults with MDD and an inadequate response to an SSRI or SNRI to vortioxetine or agomelatine (Montgomery et al., 2014). Investigators titrated the previous antidepressant down to a minimum therapeutic dose in the week before the baseline (commencement of vortioxetine or agomelatine) visit, and found the change to vortioxetine to be safe and well-tolerated (Montgomery et al., 2014). Our approach may not have been superior to a direct switch; however, we found the cross-titration was tolerated well by most study participants.

A reduction in mean total MADRS score from baseline to RCT week 2 and a further decrease from RCT week 2 to RCT week 4 were observed in the group who cross-titrated to vortioxetine as well as the group of study participants not taking an antidepressant at screening. Study participants already taking antidepressant medication at the screening visit could possibly be seen as treatment resistant, which may affect treatment outcome. Although in the first 2 weeks of the RCT this lowering of MADRS appeared to be greater in the group not taking an antidepressant at screening, this difference was not statistically significant. The subsequent decrease in mean MADRS from RCT week 2 to the RCT week 4 visit was similar in both groups. We only reported the change in MADRS up to and including the RCT week 4 visit, as any delay in onset of efficacy of vortioxetine should have emerged by this time.

Our method of cross-titration has some limitations. As stated previously, it cannot be used for individuals who are changing from a MAOI to vortioxetine. There were also small numbers of study participants changing from some antidepressant medications (tricyclic antidepressants, paroxetine, and fluvoxamine) to vortioxetine.

## Conclusion

Changing from another antidepressant to vortioxetine can be done safely while achieving treatment efficacy with vortioxetine. The cross-titration method we used to change study participants from different classes of antidepressants (selective serotonin reuptake inhibitors, serotonin noradrenaline reuptake inhibitors, tricyclic antidepressants, mirtazapine, or agomelatine) was generally well-tolerated.

## Supplementary Material

pyaa092_suppl_Supplementary_Table

## Statement of Interest

B.T.B. received speaker/consultation fees from AstraZeneca, Lundbeck, Pfizer, Takeda, Servier, Bristol Myers Squibb, Otsuka, and Janssen-Cilag.

Vortioxetine was supplied by Lundbeck without exerting any influence on the study conception, study design, data collection, data analyses, and manuscript preparation and publication. N.T.M., E.S., and C.F. have no financial interests to disclose.
