# Supplementary material for: Clinical Switching Strategies of Various Antidepressants to Vortioxetine in the PREDDICT Trial
Source: Int J Neuropsychopharmacol. 2020 Dec 3;24(4):314–21. doi: 10.1093/ijnp/pyaa092 (PMC8059490; doi:10.1093/ijnp/pyaa092)
Supplement: pyaa092_suppl_Supplementary_Table [file pyaa092_suppl_supplementary_table.docx]

**PREDDICT – Side effects**

Onset or exacerbation of side effects since last visit

| **Category** | **Specific side effect** | **Indicate if present** |
| --- | --- | --- |
| Neurological | Dizziness/Vertigo | Y / N |
|  | Headache | Y / N |
|  | Weakness/Fatigue | Y / N |
|  | Agitation/Confusion | Y / N |
|  | Collapse/Fainting | Y / N |
|  | Sedation | Y / N |
|  | Trembling and abrupt contraction of muscles | Y / N |
| Respiratory/Cardiovascular | Shortness of breath | Y / N |
|  | Chest pain/heaviness/discomfort | Y / N |
|  | Irregular heartbeat | Y / N |
| Gastrointestinal | Stomach pain | Y / N |
|  | Diarrhoea | Y / N |
|  | Constipation | Y / N |
|  | Indigestion | Y / N |
|  | Nausea/Vomiting | Y / N |
|  | Vomiting blood | Y / N |
|  | Red or black stools | Y / N |
| Skin/Mucous membranes | Skin changes – rash, itch, hives | Y / N |
|  | Infection of the mucous membranes (nose, mouth, lips, eye, genitals) | Y / N |
|  | Yellowing of the skin or eyes | Y / N |
| Systemic | Upper respiratory tract infection (sore throat, runny nose, sinusitis) | Y / N |
|  | Fever | Y / N |
|  | Influenza-type symptoms | Y / N |
|  | Swelling/Unexplained weight gain | Y / N |
| Other | Specify: | Y / N |
|  | Specify: | Y / N |
|  | Specify: | Y / N |
|  | Specify: | Y / N |
| Missed tablets since last visit | | |
| vortioxetine |  | |
| celecoxib/placebo |  | |

For each side effect ticked, please give the following details:

| **Side Effect** | **Severity** | **Onset** | **Duration** | **Relationship to study drug** | **Intervention (tick all that apply)** |
| --- | --- | --- | --- | --- | --- |
|  | - Mild - Moderate - severe | - prior to study - Since study treatment | - Resolved - Ongoing | - Unlikely - Unsure - Likely | - None - Saw doctor - Medication - Other (specify) |
|  | - Mild - Moderate - severe | - prior to study - Since study treatment | - Resolved - Ongoing | - Unlikely - Unsure - Likely | - None - Saw doctor - Medication - Other (specify) |
|  | - Mild - Moderate - severe | - prior to study - Since study treatment | - Resolved - Ongoing | - Unlikely - Unsure - Likely | - None - Saw doctor - Medication - Other (specify) |
|  | - Mild - Moderate - severe | - prior to study - Since study treatment | - Resolved - Ongoing | - Unlikely - Unsure - Likely | - None - Saw doctor - Medication - Other (specify) |
|  | - Mild - Moderate - severe | - prior to study - Since study treatment | - Resolved - Ongoing | - Unlikely - Unsure - Likely | - None - Saw doctor - Medication - Other (specify) |
|  | - Mild - Moderate - severe | - prior to study - Since study treatment | - Resolved - Ongoing | - Unlikely - Unsure - Likely | - None - Saw doctor - Medication - Other (specify) |
|  | - Mild - Moderate - severe | - prior to study - Since study treatment | - Resolved - Ongoing | - Unlikely - Unsure - Likely | - None - Saw doctor - Medication - Other (specify) |
|  | - Mild - Moderate - severe | - prior to study - Since study treatment | - Resolved - Ongoing | - Unlikely - Unsure - Likely | - None - Saw doctor - Medication - Other (specify) |
